# Robotic-Assisted Ivor Lewis Esophagectomy Is Safe and Cost Equivalent Compared to Minimally Invasive Esophagectomy in a Tertiary Referral Center

**DOI:** 10.3390/cancers16010112

**Published:** 2023-12-25

**Authors:** Sebastian Knitter, Max M. Maurer, Axel Winter, Eva M. Dobrindt, Philippa Seika, Paul V. Ritschl, Jonas Raakow, Johann Pratschke, Christian Denecke

**Affiliations:** 1 Department of Surgery, Campus Charité Mitte and Campus Virchow-Klinikum, Charité—Universitätsmedizin Berlin, Corporate Member of Freie Universität Berlin and Humboldt-Universität zu Berlin, Augustenburger Platz 1, 13353 Berlin, Germany; 2 BIH Biomedical Innovation Academy, BIH Charité Clinician Scientist Program, Berlin Institute of Health at Charité—Universitätsmedizin Berlin, Charitéplatz 1, 10117 Berlin, Germany

**Keywords:** upper gastrointestinal surgery, robotic surgery, esophagectomy, cost analysis

## Abstract

**Simple Summary:**

Recently, robotic-assisted minimally invasive esophagectomy (RAMIE) has become more common for patients with esophageal cancer. However, healthcare providers worried that RAMIE might be more expensive than the traditional minimally invasive esophagectomy (MIE). Therefore, we aimed to compare the results and costs of RAMIE and MIE in 128 patients who underwent surgery between 2017 and 2021. We found that surgical costs of RAMIE were higher. However, total costs were similar between RAMIE and MIE. Fewer cases of postoperative pneumonias were observed after RAMIE. RAMIE also tended to result in shorter hospital stays, which could explain why overall costs were about the same. All in all, our study suggests that RAMIE is not more expensive and might even be a better choice for many patients.

**Abstract:**

In recent decades, robotic-assisted minimally invasive esophagectomy (RAMIE) has been increasingly adopted for patients with esophageal cancer (EC) or cancer of the gastroesophageal junction (GEJ). However, concerns regarding its costs compared to conventional minimally invasive esophagectomy (MIE) have emerged. This study examined outcomes and costs of RAMIE versus total MIE in 128 patients who underwent Ivor Lewis esophagectomy for EC/GEJ at our department between 2017 and 2021. Surgical costs were higher for RAMIE (EUR 12,370 vs. EUR 10,059, *p* < 0.001). Yet, median daily (EUR 2023 vs. EUR 1818, *p* = 0.246) and total costs (EUR 30,510 vs. EUR 29,180, *p* = 0.460) were comparable. RAMIE showed a lower incidence of postoperative pneumonia (8% vs. 25%, *p* = 0.029) and a trend towards shorter hospital stays (15 vs. 17 days, *p* = 0.205), which may have equalized total costs. Factors independently associated with higher costs included readmission to the intensive care unit (hazard ratio [HR] = 7.0), length of stay (HR = 13.5), anastomotic leak (HR = 17.0), and postoperative pneumonia (HR = 5.4). In conclusion, RAMIE does not impose an additional financial burden. This suggests that RAMIE may be considered as a valid alternative approach for esophagectomy. Attention to typical cost factors can enhance postoperative care across surgical methods.

## 1. Introduction

In the treatment of esophageal cancer (EC) or cancer of the gastroesophageal junction (GEJ), surgical resection is crucial for long-term survival [1,2,3]. However, esophagectomy carries a high risk of complications and mortality [4]. The adoption of minimally invasive esophagectomy (MIE) has significantly improved short- and long-term outcomes compared to open esophagectomy (OE) [3,5,6,7,8]. Therefore, MIE is currently the recommended approach in the German national guidelines [9].

Since the first robotic cholecystectomy in 1994 [10] and robotic-assisted minimally invasive esophagectomy (RAMIE) in 2002 [11], robotic surgery (RS) has gained importance in various surgical specialties, including liver [12], thoracic [13] and colorectal surgery [14]. RS may offer several benefits to surgeons, such as increased freedom of movement, three-dimensional vision, and filtration of hand tremors [11,15,16]. Previous studies on the comparison between RAMIE and MIE have demonstrated comparable short- and long-term outcomes between the two approaches [17,18]. Notably, RAMIE was associated with an increased lymph node yield, that may be related to better visualization in the upper mediastinum. Hence, RAMIE may potentially be superior to MIE. However, concerns regarding the added financial burden and cost-effectiveness of RS, due to high acquisition and maintenance costs have been discussed in the literature since its introduction. Most studies have reported higher financial expenses for robotic liver surgery compared to laparoscopic or open surgery [19,20], while others have shown equal or even lower costs [21,22]. Limited data is available regarding RAMIE: So far, reports from India [23], Germany [24] and the United States [25] have demonstrated higher financial expenses for RAMIE compared to MIE. Whether these cost differences can be offset by the potential benefits of RAMIE remains unclear [26]. Still, literature lacks a detailed cost evaluation of RAMIE and MIE.

Therefore, the purpose of this study was to compare postoperative outcomes and financial expenses between Ivor Lewis RAMIE and MIE for EC/GEJ in a single-center setting. Furthermore, we aimed to identify cost drivers among patient-related and perioperative parameters.

## 2. Materials and Methods

### 2.1. Study Design

After obtaining approval from the local Institutional Review Board (Ethikkommission der Charité—Universitätsmedizin Berlin; number: EA4/052/14), we included all consecutive patients who underwent curatively intended RAMIE or MIE for EC/GEJ between 2017 and 2021 at the Department of Surgery, Campus Charité Mitte and Campus Virchow-Klinikum, Charité—Universitätsmedizin Berlin, in this single-center retrospective study. We retrospectively collected data on clinicopathological, perioperative, and financial parameters. Patients were excluded if they underwent other simultaneous surgical procedures, if surgery was palliative, or if they were under the age of 18 years at the time of resection. Patients were stratified into RAMIE or MIE groups based on the surgical approach, which was determined on a case-by-case basis considering patient wish, patient characteristics such as body-mass index (BMI), previous abdominal surgeries, or on the surgeon’s discretion. We also had to adhere to the robot’s availability as it is shared by multiple surgical teams.

### 2.2. Perioperative Management

Following our routine preoperative evaluation, adequate tumor staging, and treatment recommendation from our multidisciplinary tumor board, all patients underwent transthoracic esophagectomy (Ivor Lewis procedure) with gastric pull-up, two-field lymphadenectomy and intrathoracic anastomosis. All procedures were performed by three experienced surgeons. Preoperatively, neoadjuvant therapy included systemic chemotherapy or combined radiochemotherapy, which was not offered to some patients with severe comorbidities. All cases were discussed in our multidisciplinary tumor board according to current German S3-guidelines [9], and the indication for multimodal treatment was based on the panel’s recommendation. The patient was placed in a supine position for the abdominal part of the procedure. Double-lumen tubes were used for ventilation, and only the left lung was ventilated during the thoracoscopic part of the surgery. The procedure for patients in the MIE group was performed totally laparoscopic, and as previously reported [5]. In short, two 12 mm trocars were placed five centimeters above the umbilicus paramedially left and right. Further 12- and 5 mm trocars were put in place in the right upper abdomen, on the left costal arch and subxiphoidally. For RAMIE, the DaVinci Xi^®^ Surgical System (Intuitive Surgical Inc., Sunnyvale, CA, USA) was used. The 8 mm DaVinci optical trocar was placed through the umbilicus, and further three 8 mm DaVinci trocars were placed in a horizontal line around the umbilicus (one on the right and two on the left). A 5 mm trocar for the liver retractor was placed under the lower right rib, and a 12 mm assist trocar was placed between the right and umbilical trocar. Upon exclusion of previously unknown metastases and dissection of the lesser momentum, lymphadenectomy around the common hepatic and splenic arteries was performed. In the next step, the left gastric artery and vein were dissected and severed. The hiatus, distal esophagus and stomach were mobilized, and the omental bursa was dissected through the gastrocolic ligament. The greater curvature was mobilized up to the cardial notch. A 4–5 cm wide gastric tube was created using a 60 mm ECHELON™ Powered GST linear stapler (Ethicon Inc., Raritan, NJ, USA), beginning at the angular incisure up to the cardial notch. After the completion of the abdominal part of the procedure with the removal of all trocars and closure of all incisions, the patient was re-positioned in an over rotated left lateral position. A 4–6 cm incision was made in the posterior axillary line in the 4th intercostal space (ICS), the Alexis Laparoscopic System^®^ (Applied Medical, Rancho Santa Margarita, CA, USA) was used for temporary closure, and an 8 mm DaVinci trocar was inserted, in case of RAMIE. Further three 8 mm DaVinci trocars were inserted in the 6th, 8th, and 10th ICS between the middle and posterior axillary line. A 12 mm auxiliary trocar was placed in the 7th ICS in the anterior axillary line. For MIE, incisions of two centimeters were made in the 9th ICS and 8–10 cm dorsal from this incision. A 12 mm trocar was placed in the 6th ICS in the middle axillary line. A capnothorax of 6–8 mmHg was established in case of RAMIE. The esophagus was now mobilized laterally, beginning from the hiatus. After complete circular mobilization, the esophagus was dissected using a 60 mm linear stapler (ECHELON™) and the specimen and gastric tube were pulled into the thorax. Afterwards, the robot was disconnected, the specimen was removed via the incision in the 4th ICS, and the gastric tube was completed with additional linear staplers. For both RAMIE and MIE patients, stapled circular end-to-side intrathoracic anastomoses at the level of the azygos vein were constructed. The anastomosis was created using a circular 29 mm ECHELON™ Powered 3D stapler (Ethicon Inc., Raritan, NJ, USA), with introduction of the stapler via an incision in the stomach, that was afterwards closed with a linear stapler. Disposable instruments such as stapling devices and corresponding magazines were equivalently used between RAMIE and MIE. After surgery, all patients were routinely admitted to our specialized surgical intensive care unit (ICU), where patients were monitored for postoperative complications, such as anastomotic leak (AL) or postoperative pneumonia (PP). AL was diagnosed via endoscopy when clinically suspected (fever, elevated infectious serum parameters) or radiologically through computer tomography (CT) scans. PP was defined as new pneumonic infiltrates seen in X-ray or CT scans. Postoperative complications were graded according to the classification by Clavien and Dindo, and major morbidity was defined as grade ≥ 3a [27]. Postoperative mortality was defined as any mortality within 30 and 90 days after surgery.

### 2.3. Analysis of Financial Expenses

The controlling department of our clinic provided financial data in a cost matrix divided into various categories, including costs related to (1) surgery, (2) anesthesia, (3) intensive care, (4) dialysis if needed, (5) care on the normal ward, (6) laboratory tests, (7) cardiology, (8) radiology, (9) endoscopy, (10) other diagnostics, (11) other therapeutics including physiotherapy, and (12) patient admission. Each subdomain included costs for medical and non-medical staff, consumables, and logistics. Surgical costs included expenses for disposable instruments and sterilization of reusable instruments, respectively. The lifespan of robotic instruments was respected within the calculation as received from our institution’s controlling department. Additionally, daily and total costs per patient per stay were calculated. Acquisition costs for both the robotic and laparoscopic system were not included in the calculation. All numbers are presented in Euro (EUR). Costs were then compared between RAMIE and MIE. Furthermore, patient-, tumor- and procedure-related factors associated with increased costs were identified through multivariate analysis.

### 2.4. Statistical Analysis

Patient, tumor, perioperative and financial data were compared between RAMIE and MIE. Continuous variables were expressed as medians (range) and analyzed using the Mann–Whitney *U* test. Categorical variables were presented as frequencies and compared using the Chi-square or Fisher’s exact test, as appropriate. Increased costs were defined as costs per case exceeding the 75th percentile of the entire cohort or each group, respectively. Factors associated with increased costs were identified using a binary logistic regression model. Results were expressed as hazard ratios (HR) and 95% confidence intervals (CI) after multivariate analysis of all parameters with *p* < 0.1 in univariate analysis (Chi-square or Fisher’s exact test). Statistical significance was defined as *p* < 0.05. All statistical analyses were performed with SPSS software package for Mac OS, version 27 (IBM, Armonk, NY, USA).

## 3. Results

### 3.1. Patient Baseline Characteristics

During the study period, we identified 128 patients with EC/GEJ who underwent Ivor Lewis esophagectomy at the Department of Surgery, Campus Charité Mitte and Campus Virchow-Klinikum, Charité—Universitätsmedizin Berlin, and were included in this study based on the inclusion and exclusion criteria. RAMIE and MIE were performed in 37 (29%) and 91 cases (71%), respectively. The clinicopathological baseline characteristics are presented in Table 1. Both groups were comparable in terms of gender (*p* = 0.573), age (*p* = 0.948), BMI (*p* = 0.673), comorbidities, American Society of Anesthesiology (ASA) status (*p* = 0.920), tumor location (*p* = 0.893), T (*p* = 0.837) and N category (*p* = 0.516), UICC stage (*p* = 0.972), histologic type (*p* = 0.140), and tumor grading (*p* = 0.845). However, a significant difference was found regarding the type of preoperative therapy: More patients in the RAMIE group received chemotherapy alone, while combined radiochemotherapy was more frequently administered to patients in the MIE group (*p* = 0.045). Smoking status was equivalent between the groups (*p* = 0.264).

### 3.2. Perioperative Outcomes

Median duration of surgery was longer in the RAMIE compared to the MIE group (421 [range: 305–543] vs. 372 [range: 205–570] minutes, *p* < 0.001; Table 2). Median lengths of ICU (4 [range: 1–10] vs. 3 [range: 1–67] days, *p* = 0.528) and hospital stay (15 [range: 8–80] vs. 17 [range: 9–110] days, *p* = 0.205) were similar between the groups, although hospital stay tended to be shorter after RAMIE. There was a tendency for more red-blood cell transfusions in the MIE group, but statistical significance was not reached (0% vs. 10%, *p =* 0.058). Postoperatively, the incidence of AL was 11% after RAMIE and 14% after MIE (*p* = 0.776). However, the rate of PP was significantly lower after RAMIE (8% vs. 25%, *p* = 0.029). Postoperative overall morbidity was 38% and 54% for RAMIE and MIE, respectively (*p* = 0.101). Postoperative major morbidity was comparable between the groups (35% vs. 46%, *p* = 0.254). Readmission to ICU (19% vs. 23%, *p* = 0.606) and postoperative mortality were also similar between the groups. Oncological outcomes after surgery, including the median number of removed lymph nodes (34 [range: 22–61] vs. 32 [range: 9–72], *p* = 0.177) and the rate of positive resection margins (5% vs. 6%, *p* = 1), were comparable between RAMIE and MIE.

### 3.3. Cost Analysis and Factors Associated with Increased Costs

Details of the cost analysis are presented in Table 3. Overall, costs were mostly comparable between RAMIE and MIE. Financial expenses for anesthesia (*p* = 0.090), ICU stay (*p* = 0.236), dialysis if needed (*p* = 0.084), stay on the normal ward (*p* = 0.758), laboratory tests (*p* = 0.795), cardiology (*p* = 0.152), radiology (*p* = 0.109), endoscopy (*p* = 0.228), other diagnostics (*p* = 0.732), other therapeutics (*p* = 0.476), and patient admission (*p* = 0.625) were equivalent between the groups. However, we observed significant differences in surgical costs, with RAMIE incurring a significantly higher financial burden compared to MIE (12,370 EUR [range: 9862–19,046 EUR] vs. 10,059 EUR [range: 6589–20,170 EUR], *p* < 0.001). Still, total costs (30,510 EUR [range: 22,256–185,871 EUR] vs. 29,180 EUR [range: 18,649–303,453 EUR], *p* = 0.460) and daily costs (2023 EUR [range: 1051–4180 EUR] vs. 1818 EUR [range: 811–3365 EUR], *p =* 0.246) were comparable between the groups, indicating that higher operative costs were compensated for by overall lower costs during hospitalization.

Next, a multivariate analysis was conducted to identify factors associated with increased costs for all patients (Table 4). Increased costs were defined as total expenses exceeding the 75th percentile of the entire cohort, amounting to 42,990 EUR. In univariate analysis, the following parameters were significantly different between the groups and were subsequently entered into the multivariate analysis: readmission to ICU (*p* < 0.001), length of ICU stay ≥4 days (*p* = 0.052), length of hospital stay ≥16 days (*p* < 0.001), AL (*p* < 0.001), and PP (*p* < 0.001). The multivariate analysis identified the following parameters as independently associated with higher costs: readmission to ICU (hazard ratio [HR] = 7.0, confidence interval [CI] = 1.7–29.6, *p* = 0.008), length of hospital stay ≥16 days (HR = 13.5, CI = 1.5–118.5, *p* = 0.019), AL (HR = 17.0, CI = 2.6–109.1, *p* = 0.003), and PP (HR = 5.4, CI = 1.4–21.7, *p* = 0.017).

In cases of AL or PP, median total costs per stay increased to 64,103 EUR (*p* < 0.001) and 56,900 EUR (*p* < 0.001), respectively. For AL, increased costs were primarily associated with higher expenses for care on the normal ward (*p* < 0.001), the ICU (*p* < 0.001), endoscopy (*p* < 0.001), radiology (*p* < 0.001), laboratory tests (*p* = 0.002), and other therapeutics including physiotherapy (*p* < 0.001). In case of PP, increased financial burden was due to higher costs for care on the ICU (*p* < 0.001), dialysis (*p* < 0.001), endoscopy (*p* = 0.006), radiology (*p* < 0.001), laboratory tests (*p* < 0.001), and other therapeutics including physiotherapy (*p* < 0.001). Costs were further elevated by longer hospital stays (for AL: 49 vs. 15 days, *p* < 0.001; for PP: 36 vs. 15 days, *p* < 0.001). In case of readmission to ICU, median total cost per stay increased to 54,712 EUR (*p* < 0.001).

For RAMIE, increased costs were associated with readmission to ICU (*p* = 0.009), hospital stay ≥15 days (*p* = 0.010), and AL (*p* = 0.052) in univariate analysis. However, none of these parameters were independently associated with higher costs in multivariate analysis (Supplementary Table S1). For MIE, age ≥65 years at resection (*p* = 0.097), readmission to ICU (*p* < 0.001), length of ICU stay ≥3 days (*p* = 0.002), length of hospital stay ≥17 days (*p* < 0.001), AL (*p* < 0.001), and PP (*p* < 0.001) were identified as being associated with increased total costs in univariate analysis. In multivariate analysis, only length of ICU stay ≥3 days (HR = 20.9, CI = 1.6–278.8, *p* = 0.021) and AL (HR = 10.3, CI = 1.8–60.0, *p* = 0.009) were independently associated with higher financial expenses (Supplementary Table S2). The event of AL or PP prolonged hospital stays both for RAMIE (AL: +26 days, *p* < 0.001; PP: +21 days, *p* = 0.132) and MIE (AL: +34 days, *p* < 0.001; PP: +20 days, *p* < 0.001), while no differences could be observed between RAMIE and MIE (AL: *p =* 0.477; PP: *p* = 0.940). However, median length of stay on the ICU was only prolonged after MIE in case of PP (+4 days, *p* < 0.001; RAMIE: *p* = 0.773). Total costs were comparable between RAMIE and MIE in the event of AL (*p* = 0.477) or PP (*p* = 1).

## 4. Discussion

In this retrospective single-center study, we conducted a comparative analysis of postoperative outcomes and costs between RAMIE and MIE for EC/GEJ during the period from 2017 to 2021. Our findings revealed a longer duration of resection for RAMIE. However, the incidence of PP was significantly lower after RAMIE compared to MIE. Notably, both groups exhibited comparable oncological outcomes in terms of lymph node yield and resection margin status. In financial terms, while total costs and daily costs per stay were comparable between RAMIE and MIE, RAMIE was associated with higher costs for surgery. Multivariate analysis identified readmission to the ICU, extended hospital stay, AL, and PP as factors driving increased costs for all patients. However, only length of ICU stay and AL were independent cost drivers for MIE, while none of these factors could be identified for RAMIE.

Since the early 2000s [11], the adoption of RAMIE has been steadily increasing, but its feasibility and safety compared to MIE or OE remain topics of debate. The TIME trial by Biere et al. found that MIE resulted in reduced postoperative pulmonary infections, lower blood loss, and less pain compared to OE [1]. One-year quality of life was better after MIE, with comparable three-year overall and disease-free survival rates between MIE and OE [28,29]. Meta-analyses have confirmed the favorable postoperative outcomes associated with MIE as observed in the TIME trial, including lower blood loss, reduced overall morbidity, shorter hospital stays, and decreased pulmonary complications [2,4,30,31,32]. Long-term outcomes were equivalent, and sometimes superior, for MIE compared to OE [29,33,34]. Our retrospective study of 180 propensity-score matches patients also showed reduced postoperative morbidity and mortality for MIE [5]. Considering all available data, recent German national guidelines recommended MIE for the resection of EC/GEJ [9].

RAMIE may offer technical advantages over MIE, such as flexible instruments and three-dimensional vision in the rigid chest cavity, but current evidence supporting these benefits is limited. A Chinese RCT by He et al. comparing RAMIE and MIE for EC found comparable short-term outcomes but improved oncological outcomes with RAMIE, including more removed lymph nodes and longer recurrence-free survival [17]. Another RCT, the ROBOT trial, reported reduced overall and cardiopulmonary morbidity with RAMIE. Importantly, this study differs from our design as they performed cervical anastomoses and compared to OE [35]. Ongoing RCTs are anticipated for further insights (ROBOT-2 [36], REVATE [37]). A recent meta-analysis confirmed that RAMIE is mostly comparable to MIE regarding short-term outcomes, including intraoperative blood loss, AL, morbidity and 90-day mortality [18]. Notably, the authors reported a slight decrease in pulmonary complications, consistent with our findings. We observed a 17% reduction in PP rate after RAMIE, supporting the results of other studies [18,38,39,40,41]. Recent research lacks conclusive explanations for the improvement in PP rates. Tsunoda et al. hypothesized that a reduction in postoperative palsy of the recurrent laryngeal nerve may have played a role, which was not within the scope of our study [40]. Better visualization with the robotic system may have helped in preserving pulmonary parenchyma and nerval structures [41]. However, we observed that significantly more patients underwent combined radiochemotherapy in the MIE group, which may have influenced pulmonary morbity after surgery. Tissue damage, adhesions and a reduced lung capacity after radiochemotherapy may have increased PP rates for these patients [42,43]. Other potential contributing factors may include a slightly elevated tobacco usage and a marginally increased prevalence of preexisting pulmonary diseases in the MIE group. Given the impact of pulmonary complications on in-hospital mortality and long-term survival, preventive measures have been proposed after esophagectomy [44,45,46,47]. AL was equally diagnosed after RAMIE and MIE in our analysis, confirming the results of other studies comparing RAMIE and MIE with intrathoracic anastomoses [36,48]. Overall morbidity rates (38% for RAMIE, 54% for MIE), as well as AL rates (11% for RAMIE, 14% MIE), were within the reported numbers of 43–59% and 11–13% of recent meta-analyses, respectively [2,4,32].

While not the primary focus, RAMIE demonstrated non-inferiority to MIE regarding short-term oncological outcomes in lymph node yield and resection margins. Lymph node harvest during RAMIE varies in the literature [36,49,50], but our results, with a median of 34 removed lymph nodes, are consistent with other studies [18]. Finally, we observed a longer duration of surgery for RAMIE, which may be attributed to the learning curve as RAMIE was introduced and adopted in our department during the study period. Similar results have been reported in other studies [49,51,52]. In conclusion, our study supports RAMIE as an acceptable and promising alternative to MIE, showing mostly similar short-term postoperative outcomes, with significantly reduced PP rates.

From an economic perspective, no significant differences in total and daily costs per stay were found between RAMIE and MIE. However, further point-by-point analysis revealed higher intraoperative costs for RAMIE. All other expenses related to anesthesia and postoperative care were comparable between the two groups. The higher expenses for surgery are most likely a direct result of the prolonged duration of surgery that was observed for RAMIE. Surgical time incurs costs of approximately 40–50 EUR per minute at our institution, corresponding to the difference in surgical costs between RAMIE and MIE. Still, total costs, relating to all expenses during hospitalization, were comparable between the groups. The reduction in PP and by trend shorter length of hospital stay after RAMIE may have helped equalize total costs. In our multivariate analysis, we identified several cost drivers: Readmission to ICU, increased length of hospital stay, AL, and PP were independently associated with increased costs. These findings are expected, as the significant financial burden associated with postoperative complications and longer hospital stays or ICU readmissions is well-known for clinicians [53,54,55]. In fact, in cases of AL or PP, which are the most typical and concerning complications after esophagectomy, our cohort experienced a doubling of total costs. Further analysis revealed significantly prolonged hospital stays and increased costs in nearly all aspects of postoperative care. Notably, PP only increased ICU stay length for MIE, but not for RAMIE. These data emphasize the importance of adequate patient selection and ongoing optimization of surgical procedures and postoperative care.

Existing literature on the economic aspects of RAMIE is limited. A German review suggested higher costs for RAMIE compared to hybrid esophagectomy, but lacked statistical analysis or a detailed cost breakdown, making direct comparisons difficult [24]. A propensity-score matched study from India found significantly higher costs for RAMIE in both matched and non-matched cohorts, but also lacked a detailed cost breakdown and had a small patient sample [23]. A US study reported similar costs for RAMIE and non-robotic esophagectomy (laparoscopic or open), but the non-robotic esophagectomy group was small [25]. Therefore, to the best of our knowledge, our analysis is the first to provide a detailed financial report on RAMIE and MIE for EC/GEJ.

Our study has some limitations that should be acknowledged. Firstly, the retrospective nature of our analysis introduces inherent limitations and potential biases in patient stratification and data collection. Therefore, conclusions from our findings should be carefully drawn. Additionally, as RAMIE was implemented during the study period, a learning curve effect is to be expected, despite being performed by a small group of experienced surgeons. With further experience and proficiency in performing RAMIE, we anticipate a reduction in operative time and the incidence of postoperative complications. These improvements may also translate into cost reductions. Importantly, acquisition costs for the robotic surgical system, that are substantially higher than for the laparoscopic system, were not considered, as this would have significantly skewed the results. However, acquisition costs for the laparoscopic camera system were similarly not included in the analysis. We focused on intra- and postoperative financial expenses to identify differences potentially associated with the approaches itself. Furthermore, our study focused primarily on short-term postoperative outcomes and economic aspects, and oncologic long-term outcomes were not within the scope of our investigation, which limits a comprehensive assessment of the comparison between RAMIE and MIE. Still, we believe that our study makes a valuable contribution to the ongoing discussion surrounding the use and financial considerations of RS for esophagectomy.

## 5. Conclusions

RAMIE for EC/GEJ is associated with improved postoperative outcomes compared to MIE. From an economic point of view, the financial burden between RAMIE and MIE is comparable. Hence, RAMIE may be considered a valid alternative approach for esophagectomy. However, additional studies conducted in specialized high-volume centers are necessary to evaluate the cost-effectiveness of both approaches.

## Figures and Tables

**Table 1:** Clinicopathological data of 128 patients who underwent RAMIE or MIE for EC or cancer of the GEJ.

Characteristics	RAMIE (*n* = 37)	MIE (*n* = 91)	*p*
Male sex, *n* (%)	32 (87)	75 (82)	0.573
Median age at resection, years (range)	64 (44–81)	63 (44–82)	0.948
Age ≥ 65 years, *n* (%)	17 (46)	38 (42)	0.664
Median BMI, kg/m^2^ (range)	25.8 (15.7–36.1)	25.1 (16.1–36.4)	0.673
BMI ≥ 30 kg/m^2^, *n* (%)	4 (11)	15 (17)	0.375
Comorbidities			
Diabetes, *n* (%)	6 (16)	12 (13)	0.655
Cardiovascular disease, *n* (%)	4 (11)	9 (10)	1
Arterial hypertension, *n* (%)	20 (54)	55 (60)	0.506
Pulmonary disease, *n* (%)	4 (11)	14 (15)	0.500
Liver cirrhosis, *n* (%)	0 (0)	0 (0)	-
ASA physical status, *n* (%)			0.920
I	2 (5)	6 (7)	
II	15 (41)	37 (41)	
III	20 (54)	47 (52)	
IV	0 (0)	1 (1)	
Preoperative therapy, *n* (%)			0.045
None	2 (5)	8 (9)	
Chemotherapy	29 (78)	50 (55)	
Radiochemotherapy	6 (16)	33 (36)	
Tumor location, *n* (%)			0.893
Esophagus	20 (54)	48 (53)	
Gastroesophageal junction	17 (46)	43 (47)	
T category, *n* (%)			0.837
T0	11 (30)	26 (29)	
T1	8 (22)	20 (22)	
T2	7 (19)	14 (15)	
T3	11 (30)	28 (31)	
T4	0 (0)	3 (3)	
N category, *n* (%)			0.516
N0	22 (60)	58 (64)	
N1	6 (16)	10 (11)	
N2	5 (14)	18 (20)	
N3	4 (11)	5 (6)	
UICC stage, *n* (%)			0.972
I	20 (54)	49 (54)	
II	4 (11)	9 (10)	
III	9 (24)	25 (27)	
IV	4 (11)	8 (9)	
Lymphangiosis carcinomatosa, *n* (%)	7 (19)	16 (17)	0.741
Histologic type, *n* (%)			0.140
Adenocarcinoma	30 (81)	62 (68)	
Squamous cell carcinoma	7 (19)	29 (32)	
Tumor grading (G), *n* (%)			0.845
G1	1 (4)	3 (4)	
G2	16 (64)	42 (58)	
G3	8 (32)	28 (38)	
Smoking status, *n* (%)	16 (57)	51 (69)	0.264

RAMIE, robotic-assisted minimally invasive esophagectomy; MIE, minimally invasive esophagectomy; BMI, body-mass index; ASA, American Society of Anesthesiology; UICC, Union for International Cancer Control.

**Table 2:** Perioperative outcomes of 128 patients who underwent RAMIE or MIE for EC or cancer of the GEJ.

Characteristics	RAMIE (*n* = 37)	MIE (*n* = 91)	*p*
Median duration of resection (range), min	421 (305–543)	372 (205–570)	<0.001
Median number of lymph nodes removed (range)	34 (22–61)	32 (9–72)	0.177
Positive resection margins, *n* (%)	2 (5)	5 (6)	1
Median duration of ICU stay (range), days	4 (1–10)	3 (1–67)	0.528
Median duration of hospital stay (range), days	15 (8–80)	17 (9–110)	0.205
Need for intraoperative RBC transfusions, *n* (%)	0 (0)	9 (10)	0.058
Anastomotic leak, *n* (%)	4 (11)	13 (14)	0.776
Postoperative pneumonia, *n* (%)	3 (8)	23 (25)	0.029
Readmission to ICU, *n* (%)	7 (19)	21 (23)	0.606
Overall morbidity, *n* (%)	14 (38)	49 (54)	0.101
Major morbidity, *n* (%)	13 (35)	42 (46)	0.254
30-day mortality, *n* (%)	0 (0)	0 (0)	-
90-day mortality, *n* (%)	0 (0)	1 (1)	1

RAMIE, robotic-assisted minimally invasive esophagectomy; MIE, minimally invasive esophagectomy; ICU, intensive care unit; RBC, red blood cell.

**Table 3:** Financial data of 128 patients who underwent RAMIE or MIE for EC or cancer of the GEJ.

Parameters Costs, EUR, Median (Range)	RAMIE (*n* = 37)	MIE (*n* = 91)	*p*
Surgery	12,370 (9862–19,046)	10,059 (6589–20,170)	<0.001
Anesthesia	3375 (1691–6746)	3106 (0–9816)	0.090
ICU	4248 (548–126,105)	4981 (696–206,750)	0.236
Dialysis	0 (0–0)	0 (0–29,785)	0.084
Normal ward	6708 (3177–25,709)	6412 (705–41,230)	0.758
Laboratory tests	1684 (1080–5481)	1748 (688–12,232)	0.795
Cardiology	0 (0–2142)	0 (0–3937)	0.152
Radiology	540 (117–4671)	803 (118–8161)	0.109
Endoscopy	339 (0–14,045)	587 (0–14,024)	0.228
Other diagnostics	212 (0–384)	215 (0–1078)	0.732
Other therapeutics	355 (68–2427)	360 (0–6282)	0.476
Patient admission	0 (0–67)	0 (0–200)	0.625
Daily costs	2023 (1051–4180)	1818 (811–3365)	0.246
Total costs	30,510 (22,256–185,871)	29,180 (18,649–303,453)	0.460

RAMIE, robotic-assisted minimally invasive esophagectomy; MIE, minimally invasive esophagectomy; ICU, intensive care unit.

**Table 4:** Multivariate analysis of factors associated with increased total costs in 128 patients who underwent RAMIE or MIE for EC or cancer of the GEJ.

Parameters	UV			MV		Total Cost/Stay, EUR, Median (Range) ^%^
	<42,990 EUR per Case (*n* = 96)	≥42,990 EUR per Case (*n* = 32)	*p*	HR (95% CI)	*p*	
Male sex, *n* (%)	81 (84)	26 (81)	0.679			
Age ≥ 65 years, *n* (%)	39 (41)	16 (50)	0.354			
BMI ≥ 30 kg/m^2^, *n* (%)	15 (16)	4 (13)	1			
ASA score ≥ 3, *n* (%)	49 (52)	20 (65)	0.229			
Length of procedure ≥ 386 min ^&^, *n* (%)	51 (53)	13 (41)	0.221			
Readmission to ICU, *n* (%)	8 (8)	20 (63)	<0.001	7.0 (1.7–29.6)	0.008	54,712 (28,012–303,453)
Length of ICU stay ≥ 4 days ^&^, *n* (%)	44 (46)	21 (66)	0.052		NS	
Length of hospital stay ≥16 days ^&^, *n* (%)	37 (39)	31 (97)	<0.001	13.5 (1.5–118.5)	0.019	40,759 (19,196–303,453)
Anastomotic leak, *n* (%)	2 (2)	15 (47)	<0.001	17.0 (2.6–109.1)	0.003	64,103 (26,748–303,453)
Postoperative pneumonia, *n* (%)	10 (10)	16 (50)	<0.001	5.4 (1.4–21.7)	0.017	56,900 (19,203–303,453)

UV, univariate analysis; MV, multivariate analysis; BMI, body-mass index; ASA, American Society of Anesthesiologists; ICU, intensive care unit; NS, not significant; n/a, not applicable; ^&^, median of whole cohort; ^%^, refers to median total cost per stay in the subgroup in which the parameter applies.

## Data Availability

The data presented in this study are available on reasonable request from the corresponding author. The data are not publicly available as it contains sensitive patient information.

## Supplementary Materials

The following supporting information can be downloaded at: https://www.mdpi.com/article/10.3390/cancers16010112/s1, Table S1. Multivariate analysis of factors associated with increased total costs in 37 patients who underwent RAMIE for EC or cancer of the GEJ. Table S2. Multivariate analysis of factors associated with increased total costs in 91 patients who underwent MIE for EC or cancer of the GEJ.
