# Supplementary material for: Robotic-Assisted Ivor Lewis Esophagectomy Is Safe and Cost Equivalent Compared to Minimally Invasive Esophagectomy in a Tertiary Referral Center
Source: Cancers (Basel). 2023 Dec 25;16(1):112. doi: 10.3390/cancers16010112 (PMC10778089; doi:10.3390/cancers16010112)
Supplement: Supplementary file 1 [file cancers-16-00112-s001.zip › cancers-2631042-supplementary.pdf]

Supplementary Materials

Table S1. Multivariate analysis of factors associated with increased total costs in 37 patients who underwent RAMIE for EC or cancer of the GEJ.

Parameters	UV			MV		Total cost/stay, €, median (range)[%]
	**<36,826 € per case (n = 27)**	**≥36,826 € per case (n = 10)**	***P***	**HR (95 % CI)**	***P***	
Male sex, n (%)	25 (93)	7 (70)	0.110			
Age ≥65 years, n (%)	11 (41)	6 (60)	0.460			
BMI ≥30 kg/m^2, n (%)	3 (11)	1 (10)	1			
ASA score ≥3, n (%)	13 (50)	7 (70)	0.456			
Length of procedure ≥421 minutes[&], n (%)	13 (48)	6 (69)	0.714			
Readmission to ICU, n (%)	2 (7)	5 (50)	**0.009**		NS	
Length of ICU stay ≥4 days[&], n (%)	16 (59)	5 (50)	0.716			
Length of hospital stay ≥15 days[&], n (%)	11 (41)	9 (90)	**0.010**		NS	
Anastomotic leak, n (%)	1 (4)	3 (30)	**0.052**		NS	
Postoperative pneumonia, n (%)	1 (4)	2 (20)	0.172			

UV, univariate analysis; MV, multivariate analysis; BMI, body-mass index; ASA, American Society of Anesthesiologists; ICU, intensive care unit; NS, not significant; &, median of whole cohort; %, refers to median total cost per stay in the subgroup in which the parameter applies

Table S2. Multivariate analysis of factors associated with increased total costs in 91 patients who underwent MIE for EC or cancer of the GEJ.

Parameters	UV			MV		Total cost/stay, €, median (range)[%]
	**<47,166 € per case (n = 67)**	**≥47,166 € per case (n = 22)**	***P***	**HR (95 % CI)**	***P***	
Male sex, n (%)	56 (82)	19 (83)	1			
Age ≥65 years, n (%)	25 (37)	13 (57)	**0.097**		NS	
BMI ≥30 kg/m^2, n (%)	12 (18)	3 (14)	0.752			
ASA score ≥3, n (%)	35 (52)	14 (64)	0.351			
Length of procedure ≥372 minutes[&], n (%)	37 (54)	9 (39)	0.205			
Readmission to ICU, n (%)	7 (10)	14 (61)	**<0.001**		NS	
Length of ICU stay ≥3 days[&], n (%)	42 (62)	22 (96)	**0.002**	20.9 (1.6-278.8)	**0.021**	32,634 (19,666-303,453)
Length of hospital stay ≥17 days[&], n (%)	25 (37)	23 (100)	**<0.001**		NS	
Anastomotic leak, n (%)	2 (3)	11 (48)	**<0.001**	10.3 (1.8-60.0)	**0.009**	67,434 (26,748-303,453)
Postoperative pneumonia, n (%)	9 (13)	14 (61)	**<0.001**		NS	

UV, univariate analysis; MV, multivariate analysis; BMI, body-mass index; ASA, American Society of Anesthesiologists; ICU, intensive care unit; NS, not significant; &, median of whole cohort; %, refers to median total cost per stay in the subgroup in which the parameter applies
